# Mechanistic Principles of Hydrogen Evolution in the Membrane-Bound Hydrogenase

**DOI:** 10.1021/jacs.4c04476

**Published:** 2024-06-18

**Authors:** Abhishek Sirohiwal, Ana P. Gamiz-Hernandez, Ville R. I. Kaila

**Affiliations:** Department of Biochemistry and Biophysics, Stockholm University, Stockholm 10691, Sweden

## Abstract

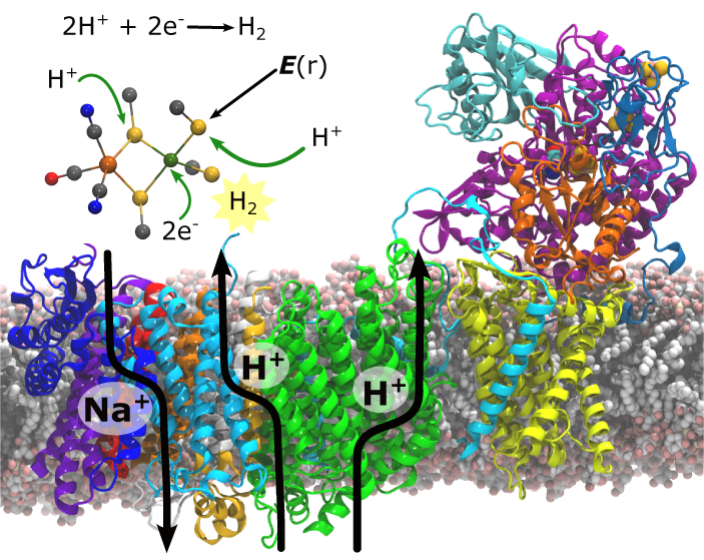

The membrane-bound hydrogenase (Mbh) from *Pyrococcus furiosus* is an archaeal member of the Complex I superfamily. It catalyzes the reduction of protons to H_2_ gas powered by a [NiFe] active site and transduces the free energy into proton pumping and Na^+^/H^+^ exchange across the membrane. Despite recent structural advances, the mechanistic principles of H_2_ catalysis and ion transport in Mbh remain elusive. Here, we probe how the redox chemistry drives the reduction of the proton to H_2_ and how the catalysis couples to conformational dynamics in the membrane domain of Mbh. By combining large-scale quantum chemical density functional theory (DFT) and correlated *ab initio* wave function methods with atomistic molecular dynamics simulations, we show that the proton transfer reactions required for the catalysis are gated by electric field effects that direct the protons by water-mediated reactions from Glu21_L_ toward the [NiFe] site, or alternatively along the nearby His75_L_ pathway that also becomes energetically feasible in certain reaction steps. These local proton-coupled electron transfer (PCET) reactions induce conformational changes around the active site that provide a key coupling element via conserved loop structures to the ion transport activity. We find that H_2_ forms in a heterolytic proton reduction step, with spin crossovers tuning the energetics along key reaction steps. On a general level, our work showcases the role of electric fields in enzyme catalysis and how these effects are employed by the [NiFe] active site of Mbh to drive PCET reactions and ion transport.

## Introduction

**Figure 1:**
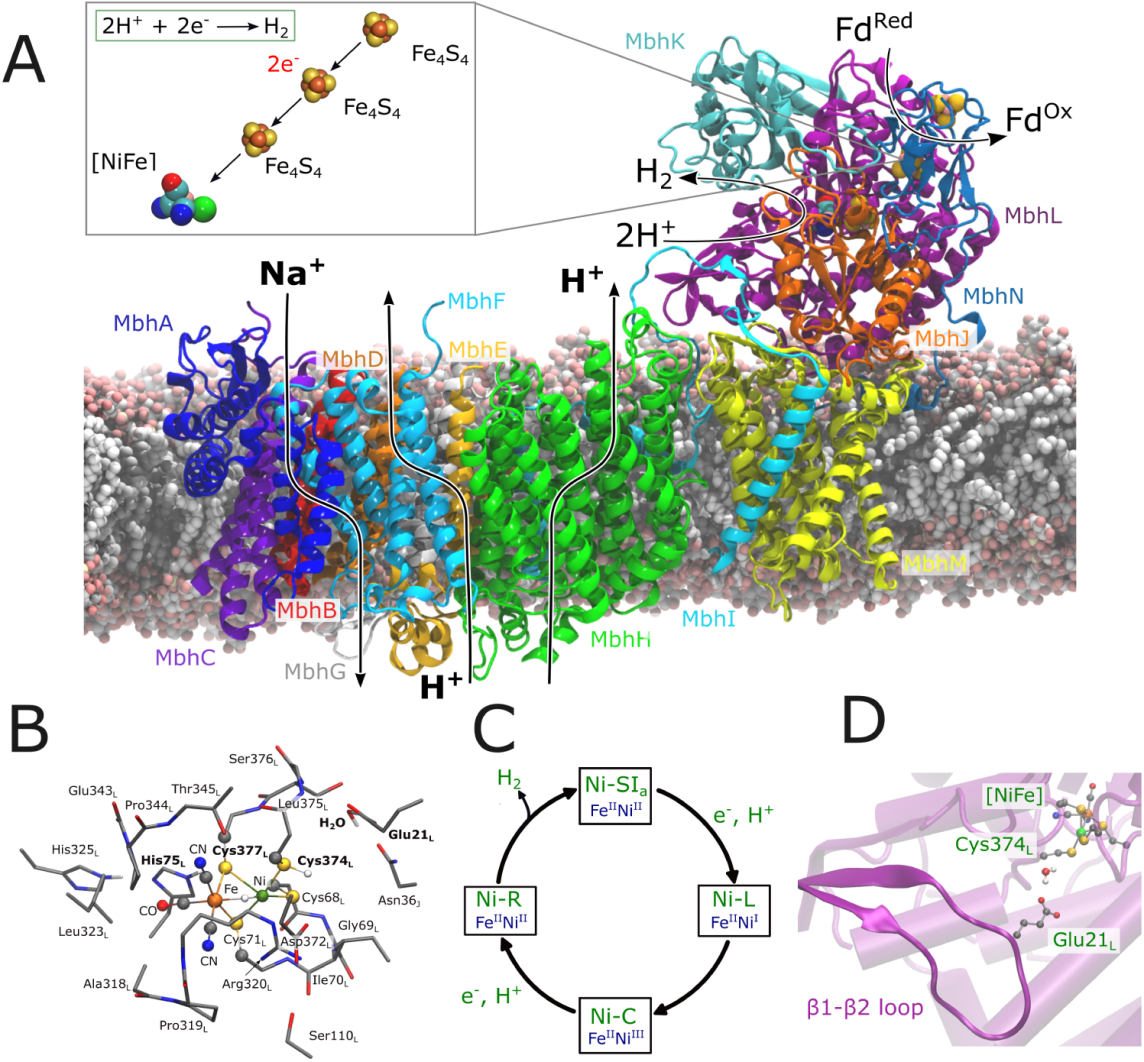
Structure, function, and catalytic cycle of Mbh. (A) Overall structure and function of Mbh, showing the hydrophilic domain, responsible for ferredoxin (Fd)-driven H^+^ reduction to H_2_ gas, and the membrane domain, responsible for proton pumping and Na^+^/H^+^ exchange across the membrane (PDB ID: 6CFW^1^). Arrows along the transmembrane region show subunits that could be responsible for the ion transport, whereas the stoichiometry and directionality are currently unknown.^5,8,10^ Redox active cofactors responsible for the electron transfer from Fd to the [NiFe] center are also shown. (B) DFT model used in the present work to study the mechanism of [NiFe] catalysis, and (C) catalytic states associated with the H_2_ catalysis in [NiFe] hydrogenases.^15^ (D) Structure of the [NiFe] active site, the β1-β2 loop, harboring Glu21_L_, and a catalytically active water molecule, hydrogen bonding with Glu21_L_ and Cys374_L_ of subunit MbhL.

The membrane-bound hydrogenase (Mbh) is an ancient enzyme found in the thermophilic archaeon *Pyrococcus furiosus* and one of the earliest members of the respiratory Complex I superfamily.^1−6^ Mbh powers the ferredoxin-driven (*E*_m_ = *ca*. −480 mV at pH = 7) reduction of protons to H_2_ (*E*_m_ = −420 mV at pH = 7),^1,7−9^ which is catalyzed by its [NiFe] active site. Mbh transduces the free energy (−60 mV/electron; −120 mV = −2.7 kcal mol^–1^ for the 2 H^+^ + 2e^–^ → H_2_ reaction) into proton pumping (H^+^) and sodium (Na^+^)/proton (H^+^) exchange, generating a sodium motive force (smf) across the biological membrane that powers the Na^+^-dependent ATP synthesis of *P. furiosus*.^10^ Mbh is a 300 kDa transmembrane protein comprising 14 subunits, with the hydrophilic domain harboring the [NiFe] active site (in MbhL) and three iron–sulfur (FeS) clusters (in MbhN, MbhJ), responsible for the electron transfer, whereas the membrane domain catalyzes Na^+^/H^+^ exchange (MbhA-G) and proton pumping (MbhM/MbhH) (Figure 1). It is closely related to the respiratory Complex I (NADH:ubiquinone oxidoreductase),^3,11^ a large (0.5–1 MDa) redox-driven proton pump that drives electron transport and oxidative phosphorylation in aerobic respiratory chains. Mbh is thus a key enzyme for understanding not only the evolution of complex bioenergetic machineries but also how H_2_ gas production powers the generation of an ion motive force across a biological membrane. Despite significant molecular understanding of soluble [NiFe] hydrogenases,^12−15^ their mechanistic principles remain puzzling and poorly understood.

For Mbh, the electrons required for the proton reduction are delivered by ferredoxin (Fd),^16^ a small (7.5 kDa) soluble electron transfer protein that docks to the MbhN subunit^2^ (Figure 1A). Fd is a one-electron donor that stepwise transfers the electrons via the three iron–sulfur clusters [4Fe-4S] located in MbhN and MbhJ to the [NiFe] center. The [NiFe] cluster (located in MbhL, Figure 1A) coordinates Cys71_L_, Cys377_L_, Cys68_L_, and Cys374_L_, and in addition, the Fe center binds two CN^–^ ligands and one CO ligand (Figure 1B). Interestingly, the [NiFe] active site has evolved into the Q-reduction site of the modern respiratory Complex I, with a striking structural resemblance together with key modular adaptations^3,11,17−19^ (Figure 1D). Based on data from soluble hydrogenases, the Ni center undergoes redox changes required for the catalysis (Ni^I^, Ni^II^, Ni^III^,^12,13,20,21^ and possibly also a Ni^IV^ form),^22^ whereas the iron remains in the Fe^II^ state during catalysis, with the diatomic strong field ligands ensuring its low spin (LS) configuration.^13−15^

Although the catalytic principles of Mbh are still poorly understood, the related [NiFe] active site in the canonical hydrogenases undergoes proton-coupled electron transfer (PCET) reactions that stepwise reduce the Ni and provide protons for the H_2_ formation reactions (Figure 1B,C).^13−15,23−25^ The Ni^II^ has a *d*^8^ electronic configuration, which can result in either a low spin (LS, *S* = 0) state with a square-planar structure or a high spin (HS, *S* = 1) state with a tetrahedral structure as in the four-coordinated Ni center in hydrogenases.^26−28^ In the experimentally characterized Ni–SI_a_^20^ and Ni–R^29^ states, the center remains in the Ni^II^ LS form, whereas the nickel is paramagnetic (*S* = 1/2) in the Ni–L^30−32^ and Ni–C^33^ states, with Ni^I^ and Ni^III^, respectively. Recently, also a high-valent LS Ni^IV^ (*S* = 0) state was assigned based on Fourier-transform infrared spectroscopy (FTIR) and electron paramagnetic resonance (EPR) spectroscopic studies in a NAD^+^-reducing [NiFe] hydrogenase.^22^ In this regard, it was proposed that the hexa-coordinated Ni^IV^ can protect the active site from O_2_-induced oxidative damage. However, despite the involvement of multiple spin and oxidation states, the role of spin crossover in H_2_ catalysis is not well understood.^26,27,34^

In addition to the unclear molecular principles underlying H_2_ formation, the pathways used for proton delivery to the active site in Mbh also remain debated. The protons required for the H_2_ evolution in the soluble [NiFe] hydrogenases are most likely transferred via a pathway involving Cys546 and Glu34 (*D. vulgarisMiyazaki F.,* hereafter *Dv*MF;^35,36^Figure S1) that connect via a hydrogen-bonding network to the bulk solvent.^15,24,36,37^ Moreover, another pathway, via His82 (His75_L_ in Mbh, His88 in *Dv*MF, also known as “histidine pathway”), was described in O_2_-tolerant membrane-bound [NiFe] hydrogenase from *Ralstonia eutropha*.^38,39^ In Mbh, four possible proton pathways lead from conserved Glu21_L_ (see below) to the [NiFe] center via water networks at the interface between MbhL/M, MbhL/I/J, MbhL/N/J, and MbhM/cleft.^5^ These putative pathways have also resemblance to proton pathways leading to the Q oxidoreduction site in Complex I,^40,41^ although their function remains unclear.

In contrast to soluble hydrogenases, the H_2_ catalysis is functionally coupled to the ion pumping activity in the membrane domain of Mbh.^1^ While the overall metal coordination has remained conserved relative to the soluble [NiFe] hydrogenases, Mbh has certain key structural differences around the [NiFe] center that could mediate the coupling effects. One such modular adaptation is established around the conserved β1-β2 loop structure (linker region Ile10_L_-Lys24_L_) of the MbhL subunit, which harbors Glu21_L_, a residue that could shuttle protons to the active site^5,42^ (see Figure 1D). The same loop undergoes conformational changes in respiratory Complex I^43−45^ and could thus trigger the proton transport activity in the membrane domain of Mbh. The distance between Glu21_L_ and the Ni-coordinating Cys374_L_ is rather large (4.8 Å) for a direct proton transfer in the cryo-EM structure of Mbh (PDB ID: 6CFW^1^), and the carboxylate side chain is poorly resolved. In this regard, recent studies^5^ suggested that Glu21_L_ could undergo a conformational change, similar to the proton shuttling Glu-242 in cytochrome *c* oxidase,^46^ and support proton transfer to the active site. Interestingly, similar structural motifs around the active site are also present in the membrane-bound formate hydrogenlyase (FHL),^6^ which also couples H_2_ formation with ion pumping. In FHL, the distance between Cys531_E_ and Glu193_E_ is around 4 Å in the cryo-EM structure (PDB ID: 7Z0S) and 7 Å in the aerobic preparation (PDB ID: 7Z0T),^6^ whereas the homologous Glu193_E_ of the soluble [NiFe] hydrogenases is located around 3.4 Å from Cys546 in the high-resolution crystal structure,^36^ and may also provide the protons for catalysis.

Here, we probe the energetics of the catalytic cycle responsible for the elusive proton reduction and H_2_ formation in Mbh by combining large-scale quantum chemical models with density functional theory (DFT), correlated *ab initio* wave function-based methods, and classical atomistic molecular dynamics (MD) simulations. Based on our multiscale approach, we identify the role of conserved residues in catalysis and how electric field effects modulate reaction barriers. Our work provides a molecular basis for understanding the link between H_2_ catalysis and the ion transport activity across the membrane domain and highlighting key differences relative to the soluble [NiFe] hydrogenase, with implications for the evolution of the Complex I superfamily. Our work also provides new insight into the [NiFe] catalysis, including the involvement of different spin states, structural assignments of Ni–R and Ni–L states, and the role of the Glu and His pathways during proton uptake that have remained much debated.

## Results

### Proton Transfer Energetics in [NiFe] Catalysis

To obtain insight into the redox-triggered proton transfer energetics in Mbh, we first constructed large quantum chemical density functional theory (DFT) models of the [NiFe] active site. The models included, in addition to the bimetallic core and its immediate ligands, all first and second sphere protein residues and water molecules obtained from our atomistic molecular dynamics (MD) simulations, leading to a molecular system with around 260 atoms (see Figure 1B and *Methods*). It should be noted that the water molecule between Cys374_L_ and Glu21_L_ is not resolved in the cryo-EM structure of Mbh. Additionally, it is absent in the structure of soluble hydrogenases, as Cys546 and Glu34 form a direct hydrogen bond.^36^ Based on results from our current detailed MD simulations (see Figure 4A–C) as well as our previous work,^5^ we have positioned a water molecule between Cys374_L_ and Glu21_L_, where it shows an extended residence time in the MD simulations. Based on the DFT models, we further optimized reaction pathways for proton transfer leading to H_2_ formation along the four experimentally characterized redox states (Ni–SI_a_, Ni–L, Ni–C, and Ni–R) of the catalytic cycle (see Figure 1C). Our extensive benchmarking calculations relative to high-resolution (0.89 Å) X-ray data^36^ as well as *ab initio* wave function theory (RPA, DLPNO–CCSD(T_1_)) show that the employed DFT methodology provides an accurate description of the geometry, electronic structure, and reaction energetics of the [NiFe] site (Figures S1–S3, Tables S1–S4).

**Figure 2:**
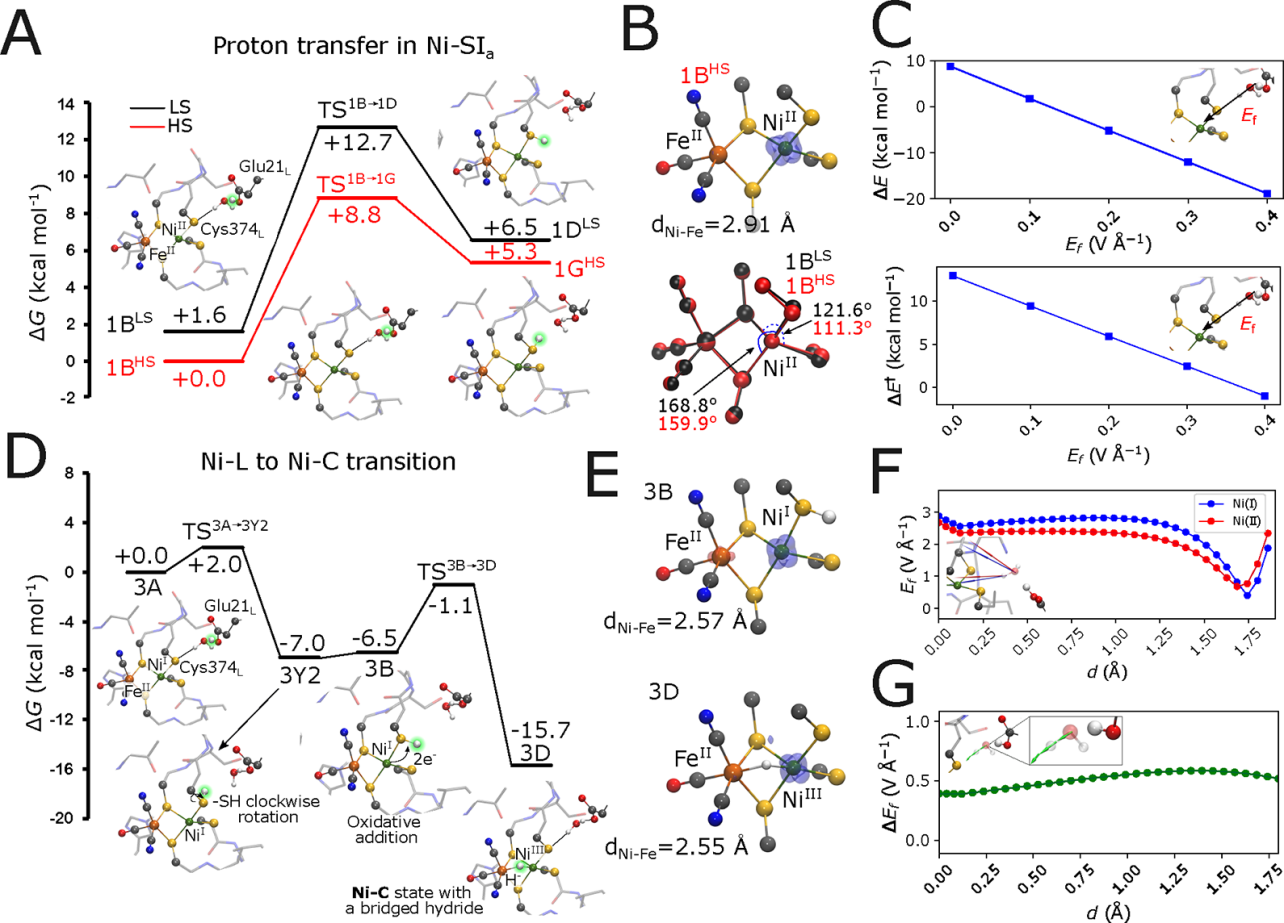
Reaction energetics in the Ni–SI_a_, Ni–L, and Ni–C states. (A) Energetics of proton transfer from Glu21_L_ to Cys374_L_ in the low spin (LS) and high spin (HS) forms of the Ni–SI_a_ state. The transferred proton is highlighted in green. (B) Top: spin density distribution in the **1B**^**HS**^ state, and bottom: overlay of optimized LS and HS models of the Ni–SI_a_ state. (C) Dependence of the reaction energy and barrier on an electric field applied along the proton transfer coordinate. (D) Energy profile for proton transfer from Glu21_L_ to Cys374_L_ in the Ni–L state and subsequent transition to the Ni–C state. The transferred proton is highlighted in green. (E) Spin density distribution in the Ni–L (**3B**) and Ni–C (**3D**) states, with marked Ni–Fe distances. (F) Electric field strength along the H_2_O–Cys374_L_ proton transfer coordinate in the Ni–SI_a_ state (**1B**^**LS**^) and in the one-electron-reduced Ni–L state. *Inset*: the electric field vectors along the proton transfer coordinate for the Ni–SI_a_ (Ni^II^) and Ni–L states (Ni^I^). (G) Electric field difference in the Ni–SI_a_ minus Ni–L states. *Inset*: the electric field vectors point toward the sulfur atom of the Cys374_L_ upon reduction of Ni^II^.

We first explored the structure of the [NiFe] in the Ni–SI_a_ state, with the metal core modeled in the Fe^II^/Ni^II^ configuration, with the nickel in the singlet (*S* = 0, LS) or triplet (*S* = 1, HS) state (Figure 2A). To obtain insight into the proton transfer energetics, we permutated the proton on the possible protonatable residues around the active site (Glu21_L_, Cys374_L_, Cys377_L_, Cys68_L_, and His75_L_) and optimized reaction pathways for the proton transfer reaction between the different sites (Figures S5–S7 and Tables S5–S7). Overall, the optimized geometries (Figures 2B, S5, S6) suggest that the coordination environment around Ni^II^ in the low- and high-spin forms is almost identical with “see-saw”-like coordination (Figure 2B); this could arise from the restricted movement of ligands due to structural constraints imposed by the protein scaffold. However, we observed subtle differences in the metal–ligand bond lengths between the spin states (e.g., Ni–S_C377_ is 2.19 and 2.38 Å in **1B**^**LS**^ and **1B**^**HS**^, respectively). See Table S6 for a detailed analysis).

The water-mediated proton transfer from Glu21_L_ to Cys374_L_ has a modest free energy barrier (Δ*G*^‡^= +12.7 kcal mol^–1^ in LS, Δ*G*^‡^= +8.8 kcal mol^–1^ in HS), but protonation of Cys374_L_ is energetically unfavorable (Δ*G* = +6.5 kcal mol^–1^ in **1D**^**LS**^; + 5.3 kcal mol^–1^ in **1G**^**HS**^) (Figure 2A, see also Figure S7). When Glu21_L_ is protonated, both spin states are nearly isoenergetic (ΔΔ*G* = 1.6 kcal mol^–1^), with a small overall preference for the HS form (Figure 2A). We find that the proton transfer from Glu21_L_ to Cys68_L_ (+15.6/+15.4 kcal mol^–1^ for **1E**^**LS**^**/IE**^**HS**^**)** or from His75_L_ to Cys377_L_ is also energetically highly unfavorable (Figures S5, S6). The energetics compares well with our wave function-based *ab initio* calculations, suggesting that our hybrid density functional treatment captures the reaction energetics of the system within an overall accuracy of a few kcal mol^–1^ for the electronic effects (Tables S3, S4). Taken together, these calculations show that the overall proton transfer from Glu21_L_ or His75_L_ to the cysteine ligands or to the metal site in the Ni–SI_a_ state is unfavorable (see also Table S6 for structural analysis).

One-electron reduction of Ni–SI_a_ leads to the Ni–L state, with a Ni^I^/Fe^II^ (*S* = 1/2) configuration, with the reduction strongly favoring the proton transfer toward the [NiFe] cluster (Figures 2D, S8, S9, Movie S1). In this regard, the proton transfer from Glu21_L_**(3A)** to Cys374_L_**(3Y2)** becomes strongly exergonic upon formation of the Ni–L state (Δ*G* = -7.0 kcal mol^–1^) with a small reaction barrier (Δ*G*^‡^ ∼ 2.0 kcal mol^–1^, Figure 2D). The rotation of the protonated thiol of Cys374_L_ toward the [NiFe] site is nearly isoenergetic (see below, **3B**, −6.5 kcal mol^–1^; ∼92° clockwise with respect to −SH group in **3Y2**, see Figure 2D), with a two-center Ni–Fe metal–metal bond (Figure 2E, Tables S8, S9) forming at the core (*cf*. also ref. (31)). 3B and 3Y2 differ only in the rotated thiol group of Cys374_L_, with both states featuring a short Ni–Fe distance of 2.57 Å. The proton transfer to Cys68_L_ (**3G1**, −4.1 kcal mol^–**1**^, Figure S8) is also exergonic, suggesting that the proton could populate different rotameric configurations of both Cys374_L_ and Cys68_L_, consistent with the experimentally observed multiple forms of the Ni–L state.^47,48^ However, in contrast to the energetically favored Glu21_L_-mediated pathway, we find that His75_L_-mediated proton transfer to the cluster is strongly endergonic (Figure S8) in the Ni–L state. Taken together, our findings suggest that the initial proton transfer reaction energetically favors the glutamate pathway (but see below).

Subsequent oxidation of the Ni center forms a Ni^III^/Fe^II^ (*S* = 1/2) configuration, known as the Ni–C state. This reduces the transferred proton to a hydride (H^–^) ion, mediated by a *metal*-to-*ligand* electron transfer between the Ni and the H^+^ (Figures 2D, S8, S9, Tables S8, S9). Protonation of the metal-core involves further rotation of the Cys-H bond, allowing the H^–^ to coordinate to the bridging (μ-H^–^) position (**3B** →**3D,** Δ*G*^‡^ = +5.4 kcal mol^−1^ with both the Ni^III^ and Fe^II^ (*d*(Ni–H^–^) = 1.60 Å/*d*(Fe–H^–^) = 1.70 Å, Figure S9, Movie S2). The established state closely resembles the experimentally characterized Ni–C form of the soluble [NiFe] hydrogenases.^49^ In contrast, we find that the formation of a terminal Ni–H^–^ configuration (*d*(Ni–H^–^) = 1.44 Å/*d*(Fe–H^–^) = 3.54 Å) is energetically unfavored (Figure S8, see also Table S9 for geometric analysis). The free energy profiles thus suggest that the conversion of Ni–L to Ni–C state is exergonic (**3Y2** → **3D**, Δ*G* = -8.7 and Δ*G*^‡^ = +5.8 kcal mol^−1^), and support the progression of the reaction toward the reduced metal-bridged (Ni–H^–^–Fe) hydride state (Figure 2D, E), but the additional protonation of the cysteine ligands is energetically unfavored in this state (Figures S10, S11), preventing further proton uptake in the Ni–C state (see below).

### Electric Field-Induced Proton Transfer Reactions

To probe the molecular basis for the redox-triggered change in reaction barriers and the thermodynamic driving force for proton transfer in the Ni–SI_a_ → Ni–L transition, we quantified electric field (***E***) effects around the active site. Our calculations suggest that the formation of the reduced Ni–L state increases the electric field along the proton pathway leading from Glu21_L_ to Cys374_L_ by |Δ***E***_f_| = 0.6 V Å^–1^ relative to the oxidized Ni–SI_a_ state, with the reduction resulting in an electric field vector that points along the proton pathway toward Cys374_L_ (Figure 2F, G), with a stronger field effect relative to other coordinating residues (Figure S26). To further test how the electric fields could modulate the reaction barriers, we applied an external field in the direction of the H_2_O → Cys374_L_ bond in the Ni–SI_a_ state. Interestingly, this applied field similarly lowers the reaction barriers and reaction energy with a linear shift with increasing field strengths that closely resembles the effects observed upon formation of the Ni–L state. These findings suggest that redox-triggered electric field effects could drive the proton transfer toward the [NiFe] center (Figure 2C) by similar effects as observed for other energy-transducing systems, such as cytochrome *c* oxidase,^50^ Complex I,^51^ and Photosystem II^52^ (*cf*. also ref. (51)).

### Mechanism of H_2_ Formation in the Ni–R State

Our DFT calculations suggest that the further one-electron reduction of Ni–C leads to a Ni^II^/Fe^II^ configuration, known as the Ni–R state, with energetically accessible LS and HS forms. To probe the thermodynamic and kinetic feasibility of transferring the second proton required for the H_2_ formation in this state, we studied the energetics of proton transfer from both Glu21_L_ and His75_L_ toward the hydride bridging the [NiFe] core, and based on these, we explored the energetics of forming H_2_ within the [NiFe] core.

**Figure 3:**
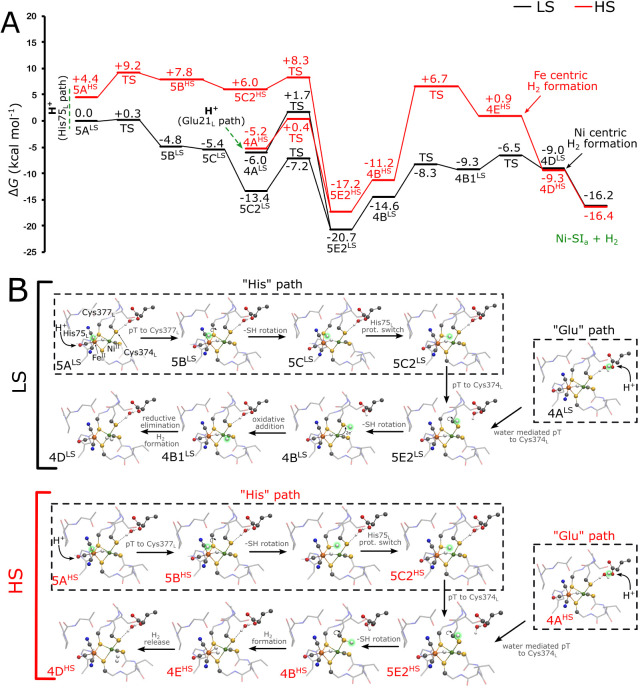
Free energy profile of H_2_ formation in the Ni–R state. (A) Free energy profile of the H_2_ formation in the low spin (LS, in black) and high spin (HS, in red) configurations of the Ni–R state. Energetics of proton transfer via the histidine (His75_L_) and glutamate (Glu21_L_) pathways. (B) Structure of different intermediates involved in the H_2_ formation step. His75_L_ can remain protonated (His^+^), while the Glu pathway is used for the second proton delivery (see Figures S23–S25). The structures of the transition states are shown in Figuress S14–S15.

We find that the proton transfer from Glu21_L_ (**4A**^**LS/HS**^) to Cys374_L_ (**5E2**^**LS/HS**^) becomes strongly downhill in the Ni–R state (Δ*G* = −14.7/–12.0 kcal mol^–1^ in LS/HS, Figure 3), whereas the proton transfer to Cys68_L_ is endergonic (by ca. Δ*G* = +5.2/+3.2 kcal mol^–1^, see Figures S12–S16, Tables S10–S12). The protonation of Cys374_L_ has a small reaction barrier (**4A**^**LS/HS**^ → **5E2**^**LS/HS**^, Δ*G*^‡^= +7.7/+5.6 kcal mol^–1^ for LS/HS), which supports the fact that the reaction is kinetically feasible. Moreover, due to the small energy gap between the spin states, the proton transfer step could employ spin crossover between the LS and HS configurations, which in turn would reduce the reaction barriers along the HS pathway (Figure 3). It is to be noted that the reaction barrier for the LS pathway is significantly reduced when His75_L_ is protonated (see below). Similarly, as for the first proton transfer step (see above), we find that the forward progression of the reaction involves rotation of the thiol group of Cys374_L_ (**5E2**^**LS/HS**^ → **4B**^**LS/HS**^, Δ*G* = +6.1/+6.0 kcal mol^–1^ for LS/HS) toward the metal-bound hydride. From here, the H_2_ formation takes place by the oxidative addition at the Ni^II^ center, thereby forming a dihydride-bound Ni^IV^/Fe^II^ species in the LS state (**4B1**^**LS**^, Movie S3, **4B**^**LS**^ → **4B1**^**LS**^, Δ*G*^‡^*=* + 6.3 kcal mol^–1^), with an octahedral coordination around a putative Ni^IV^ state. Subsequent reduction of the Ni^IV^ to Ni^II^ (**4B1**^**LS**^ → **4D**^**LS**^, Δ*G* = +0.3, Δ*G*^‡^*=* +2.8 kcal mol^–1^) couples to the formation of the H_2_ in the Ni^II^ form **(4D**^**LS**^, Ni–H_2_ is 1.59 Å). In contrast, the H_2_ formation along the HS surface is highly endergonic (**4B**^**HS**^ → **4E**^**HS**^, Δ*G* = +12.2, Δ*G*^‡^ = +17.9 kcal mol^–1^, Figure 3) and requires a large rotational movement of the Cys374_L_ thiol group toward the bound hydride, with H_2_**(4E**^**HS**^**)** forming at the bridging position (*d*(Ni–H_2_) = 2.08 Å, *d*(Fe–H_2_) = 1.68 Å).

Notably, the high-resolution structure of the [NiFe] hydrogenase from *Dv*MF in the Ni–R state^36^ also shows a bridging hydride and a protonated Cys374_L_ (referred to as Cys546 in *Dv*MF), with a shorter Ni–H^–^ bond (1.58 Å) relative to the Fe–H^–^ bond (1.78 Å). This state closely resembles our energetically favored LS form of the Ni–R state (**5E2**^**LS**^, Ni–H^–^/Fe–H^–^ are 1.58 Å/1.70 Å), suggesting that the H_2_ formation is strongly favored along the LS pathway.

### Involvement of the Histidine Pathway in H_2_ Formation

To probe the possible role of His75_L_ in both H_2_ formation and proton delivery,^38,39^ we also optimized all putative states along the catalytic cycle, but with His75_L_ modeled in its protonated (HisH^+^) form (Figures S17–S25, Tables S14–S21). For the Ni–R state, we find that the proton transfer from His75_L_**(5A**^**LS**^**)** to Cys377_L_**(5B**^**LS**^**)** is exergonic and has a small reaction barrier (**5A**^**LS**^ → **5B**^**LS**^, Δ*G* = -4.8, Δ*G*^‡^=+0.3 kcal mol^–1^, Figure 3). Tilting of the Cys377_L_**(5C**^**LS**^**)** allows the −SH group to move away from the bridging position (**5B**^**LS**^ → **5C**^**LS**^, Δ*G* = −0.6 kcal mol^–1^) that leads to a subtle shift in the side chain of His75_L_ (Figure 3B). The proton transfer from Cys377_L_**(5C2**^**LS**^**)** to Cys374_L_**(5E2**^**LS**^**)** is exergonic (Δ*G* = −7.3 kcal mol^–1^) with a small reaction barrier (Δ*G*^‡^ = +6.2 kcal mol^–1^) (Figures S12–S14), while the subsequent proton transfer from Cys374_L_ toward the hydride is also energetically feasible along the same mechanism as discussed above **(5E2**^**LS**^ → **4B**^**LS**^ → **4B1**^**LS**^ → **4D**^**LS**^**)**. Interestingly, the proton transfer along this pathway is exergonic in the LS configuration but endergonic in the HS state (**5A**^**HS**^ → **5B**^**HS**^, Δ*G* = +3.4 kcal mol^–1^ and Δ*G*^‡^ = +4.8 kcal mol^–1^, Figures 3, S14–S16). As for the Glu pathway, our analysis suggests that electric field effects could also play an important role in barrier modulation for the His pathway (Figure S26).

In addition to its possible role as a proton uptake pathway, we found that the protonation of His75_L_ modulates the proton transfer energetics along the Glu pathway. In this regard, the proximity of the positively charged HisH^+^ next to the [NiFe] center leads to an increase in proton transfer barriers for Glu21_L_ to Cys374_L_ in the Ni–SI_a_ to Ni–L transition (Figures S17–S21), as well as an increase in the reaction barrier for hydride binding during the Ni–L to Ni–C transition (see also Figure S21). In contrast, the proton transfer barrier from Glu21_L_ to Cys374_L_ decreases by +5.4 kcal mol^–1^ in the LS Ni–R state (Figures S23–S25). The latter effect could arise from a directional electric field (Δ*E*_f_ = 0.1 V Å^–1^) formed along the Glu21_L_-Cys374_L_ pathway upon the protonation of His75_L_ (Figure S26). We find that the His75_L_ protonation favors the HS over the LS form in the Ni–SI_a_ state (Figure S19), but does not affect the spin energetics in the other redox states (Figure S25). Taken together, these findings suggest that protonation of His75_L_ could act as a pH switch that modulates the reaction energetics, consistent with altered [NiFe] activity dependence on the pH and its possible functional “pH sensing”.^32^

Taken together, our findings demonstrate that the second proton transfer required for H_2_ catalysis could occur via either the “Glu pathway” (in both LS and HS configurations) or the “His pathway” (in the LS configuration). The latter requires protonation of His75_L_, suggesting that the His pathway could be active at low pH upon formation of the Ni–R state and regulate the activity of the [NiFe] center.

### H_2_ Release and Active-Site Recovery to Ni–SI_a_

Our DFT calculations suggest that the binding of H_2_ in the Ni–R state is modulated by the spin state. In the LS form (**4D**^**LS**^), H_2_ prefers binding in a side-on, *η*^*2*^ configuration to Ni^II^, whereas H_2_ binds in a bridging position between Ni and Fe in the HS configuration (**4E**^**HS**^), although the state is energetically highly unfavored (Δ*G*^HS-LS^ = +10 kcal mol^–1^, Figure 3A). However, dissociation of H_2_ leads to crossing of the spin states (**4D**^**LS**^**→ 4D**^**HS**^, Δ*G* = +0.3 kcal mol^–1^), and results in a degenerate *apo*-Fe^II^Ni^II^ state for both the HS and LS forms (Δ*G* = +0.2 kcal mol^–1^). These findings suggest that H_2_ formation and dissociation could involve spin crossover. We find that the H_2_ dissociation is coupled with a rather large entropic (*T*Δ*S*) effect of −9.3 kcal mol^–1^ that could be relevant for transducing the free energy for ion transport (Figure S13). Our calculations further show that the release of H_2_ restores the Ni–SI_a_ state.

### Redox-Triggered Conformational Changes Trigger the Ion Transport Machinery

In order to probe the conformational dynamics coupled to catalysis, we performed atomistic molecular dynamics (MD) simulations of Mbh embedded in a membrane–water–ion environment to probe how the redox-coupled proton transfer reaction induces conformational and hydration changes (Tables S22, S23). In this regard, we derived atomistic force field parameters based on the quantum chemical models for the [NiFe] cluster along the key steps of the catalytic cycle which allowed us to study the coupling between the redox catalysis and the conformational dynamics.^5^

**Figure 4:**
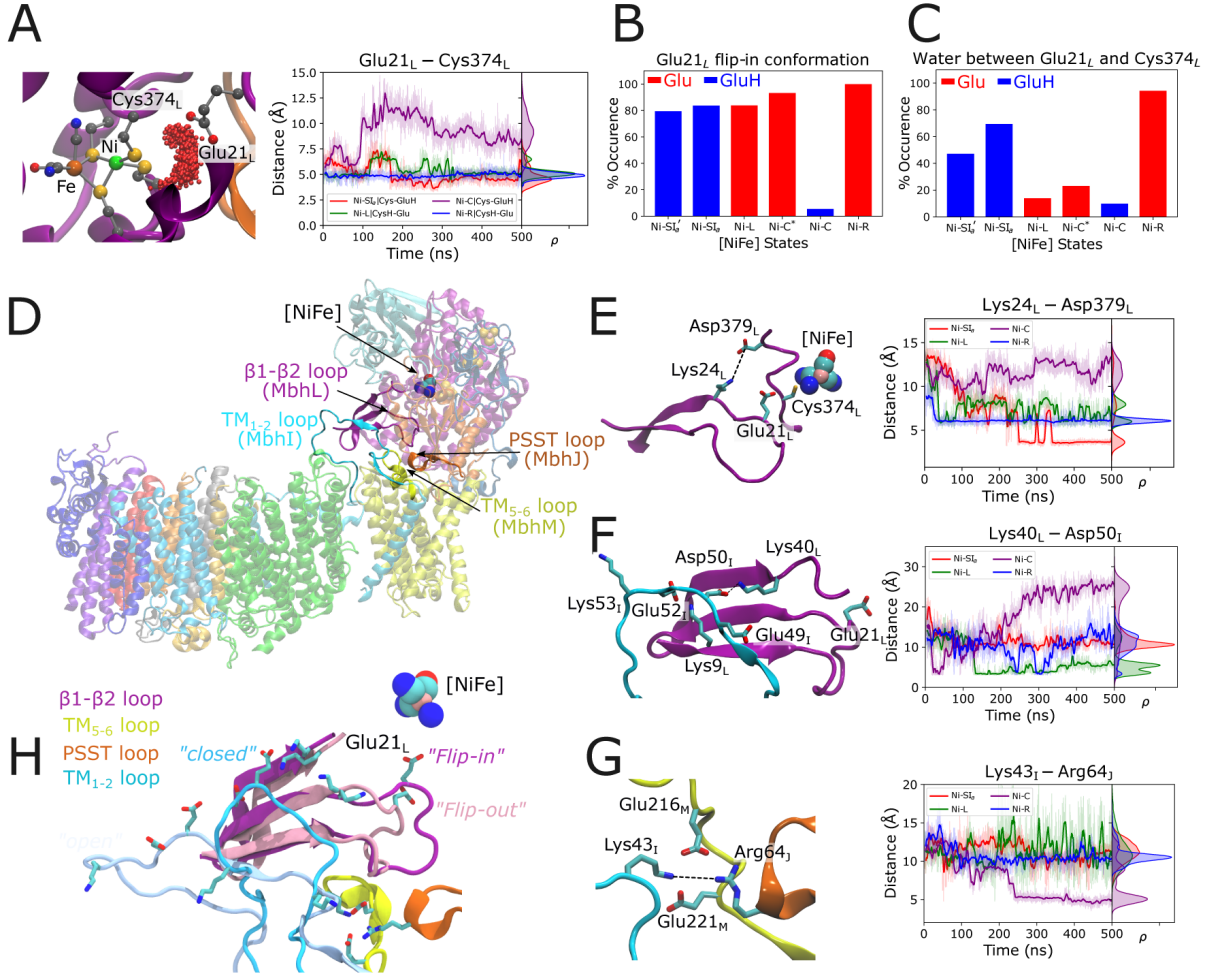
Redox-triggered conformational coupling between redox catalysis and loop dynamics. (A) Clustering of water molecules between the Glu21_L_ and the [NiFe] cluster, and the distance between Cys374_L_ and Glu21_L_ in different catalytic states from MD simulations. (B) Population of “flipped-in” conformation of Glu21_L_ in different catalytic states. Ni–SI_a_’ and Ni–SI_a_ differ in the modeled redox states of the Fd and Fe–S clusters (see Table S23), while in the Ni–C* state, Glu20_L_ is protonated and Glu21_L_ deprotonated (and vice versa in the Ni–C state). (C) Water occupancy between Glu21_L_ and Cys374_L_ in MD simulations. (D) Structure and location of the loop network and the [NiFe] cluster of Mbh. (E) Effect of the Glu21_L_ “flip” on the ion-pair dynamics in different catalytic states. The outward motion of Glu21_L_ in the Ni–C state favors dissociation of the ion pair with Lys24_L_ and Asp379_L_, and contraction of the β1-β2 loop (see panel H). (F) Interaction of the TM1–2 loop (MbhI) and the β1-β2 loop (MbhL). The contraction of β1-β2 leads to dissociation of an ion-paired network between different subunits and an outward motion of the TM1–2 loop (see also panel H). (G) Interactions among the TM1–2 loop, the TM5–6 loop, and the PSST loop. The TM1–2 loop forms an ion pair among Lys43_I_, Glu216_M_, Glu221_M_, and Arg64_J_. (H) Conformational changes in loops upon conformational changes in Glu21_L_ (“inward”–dark colors; and “outward”–light colors orientations). Subsequent conformational changes in the TM1–2 loop are also shown (“open” vs “close”). Details of the redox and protonation states in various states are shown in Tables S22 and S23.

Our MD simulations suggest that reduction of the [NiFe] center changes the conformational dynamics of Glu21_L_, and leads to an increase in the “flipped-in” conformation of the residue. This increases the occupancy of the proton transfer-mediating water molecule, bridging the Glu21_L_– Cys374_L_ gap during Ni–SI_a_ → Ni–L and Ni–C → Ni–R transitions (Figure 4).^5^ Interestingly, the proton transfer from Glu21_L_ to Cys374_L_ induces a conformational change, which leads to dissociation of the water molecule and favors the outward flipping of the anionic Glu21_L_ (Figure 4A–C), which could prevent back transfer of the proton from Cys374_L_ to Glu21_L_/bulk solvent but also provide a possible coupling element that triggers ion transport in the membrane domain of Mbh (see below). Recent cryo-EM structures of the FHL complex^6^ also show a cavity between Cys374_L_ and Glu21_L_ in one structure (PDB ID: 7Z0T, with a distance of around 7 Å) that could occupy a similar proton transfer-mediating water molecule, but not in an anaerobic sample (PDB ID: 7Z0S, with a distance of around 4 Å). Moreover, this feature has not been reported for soluble [NiFe] hydrogenases (*Dv*MF). However, we note that although Cys546 and Glu34 form a strong hydrogen-bonding interaction in *Dv*MF, FTIR experiments^24^ suggest that a “dangling” water molecule realigns upon changes in protonation states during the Ni–C → Ni–L transition. The outward flip of the protonated Glu21_L_ in our simulations of the Ni–C state further stresses the possible functional role of His75_L_ in providing a second proton for the H_2_ formation (Figure 3B). Sequence and structure comparison with the Complex I superfamily (Figure S30) show that Glu21_L_ occupies the same position as His38^Nqo4^ (*T. thermophilus* numbering), which is likely to function as a proton donor in the quinone reduction process in Complex I^40^ (cf. also.^53−55^).

We next analyzed how the redox and protonation changes in the active site could activate the ion transport activity across the membrane by probing conformational changes in the membrane domain and surrounding loop structures. In this regard, it was recently suggested^44^ that the quinone reduction in Complex I couples to conformational changes in the surrounding loop regions that could trigger a π-to-α transition in the transmembrane helix (TM3^ND6^), enabling proton transport in the membrane domain. Interestingly, many of these loop regions are also conserved in Mbh (Figures 4D–H, S28). Our MD simulations suggest that loop regions of β1-β2 (MbhL) and TM1–2 (MbhI) undergo conformational changes during the Ni–C → Ni–R transition (Figures 4, S27, S28). In the Ni–C state, we observe an outward flip of the protonated Glu21_L_ and a contraction of the β1-β2 loop, which results in the dissociation of the Lys24_L_-Asp379_L_ ion pair. This conformational change destabilizes an ion-paired network involving Lys40_L_, Lys9_L_, Glu49_I_, and Asp50_I_, similarly as in Complex I.^44^ Interestingly, when the MD simulations are performed in other catalytic states (Ni–SI_a_, Ni–L, Ni–R), we observe stable interactions between the loop region of TM1–2 and β1-β2. Destabilization of the ion-paired network between the TM1–2 and the β1-β2 loop in the Ni–C state leads to a large-scale conformation change of the TM1–2 loop that propagates toward the membrane arm (Figure 4H), consistent with the high B-factors and blurred density of the TM1–2 loop observed in the cryo-EM data.^1^ Despite common redox-driven electrostatic and conformational changes that resemble the coupling elements suggested for Complex I, we find that many of the charged residues in TM1–2 (Glu49_I_, Glu52_I_, Lys53_I_) are unique to Mbh. Moreover, the region is more hydrophobic in Complex I relative to MbhM–a feature that could have evolved to support binding of the nonpolar quinone substrate.

## Discussion

In this work, we have proposed a molecular mechanism of the ancient membrane-bound hydrogenase, which reduces protons to form H_2_ gas and couples this redox chemistry to ion pumping across the archaeal membrane. To this end, we derived an electronic structure-level understanding of the H_2_ formation at the [NiFe] active site and probed how the redox-driven large-scale conformational changes in loops connect the electron transfer domain to the membrane module.

**Figure 5:**
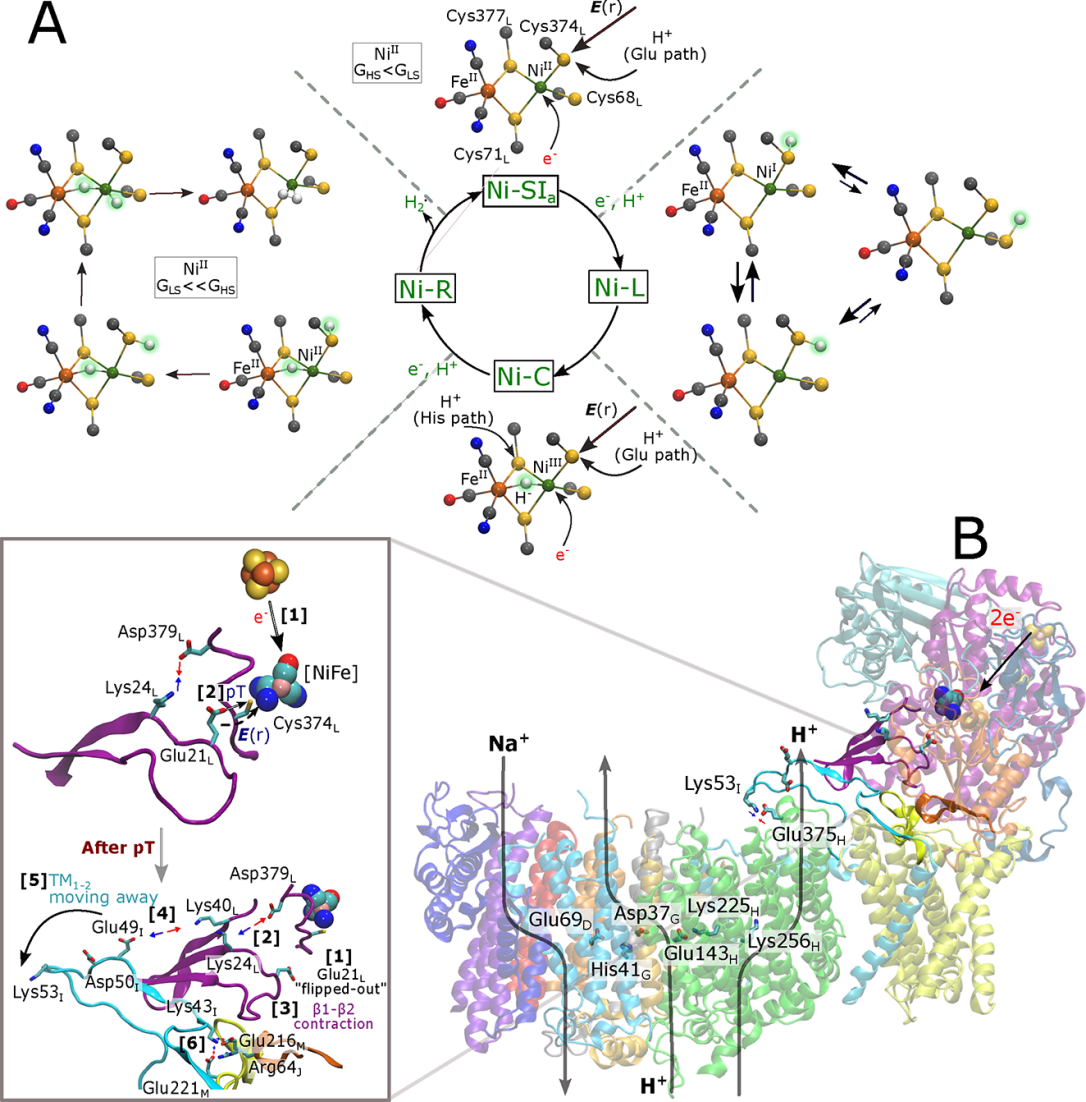
Structural models of intermediate states in the catalytic cycle and proposed coupling elements. (A) Structural models for the Ni–SI_a_, Ni–L, Ni–C, and Ni–R states in Mbh. The catalysis proceeds via the one-electron reduction of Ni–SI_a_ and the subsequent generation of a directed electric field gradient that drives the proton transfer from Glu21_L_ to Cys374_L_/Cys68_L_, forming the Ni–L state. The high spin (HS) state is more stable relative to the low spin (LS) form for the Ni–SI_a_ state (*G*_HS_ < *G*_LS_). Different structures exist for Ni–L, with energetically degenerate rotamers of the protonated Cys374_L_ or protonated Cys68_L_. Subsequent oxidation of Ni^I^ leads to the Ni–C state (Ni^III^) with a bridging hydride (μ-H^–^). Subsequent one-electron reduction of the Ni–C state forms the Ni–R state (Ni^II^), followed by proton transfer along the Glu or His pathways that result in the protonation of Cys374_L_ or Cys377_L_, respectively. Possible representative structures of the Ni–R state are shown along the H_2_ formation reaction (see also Figure 3). H_2_ formation in the Ni–R state is favored by the LS Ni^II^ form (*G*_LS_ ≪ *G*_HS_). (B) Suggested redox-driven long-range coupling elements in Mbh. *Top inset:* Primary signal transduction steps induced by the [NiFe] redox chemistry involve: [1] one-electron reduction of the [NiFe] by the proximal FeS cluster, which induces a directed electric field toward Cys374_L_ that [2] leads to proton transfer from Glu21_L_ to Cys374_L_. The “flipped-in” orientation of Glu21_L_ favors the proton transfer and formation of an ion-paired interaction between Lys24_L_ and Asp379_L_. *Bottom inset:* Following the proton transfer step: [1] Glu21_L_ flips into its “outward” conformation, which [2] favors dissociation of the Lys24_L_-Asp379_L_ ion pair. Subsequently, the β1-β2 loop undergoes a contraction [3], which in turn favors the dissociation of ion pairs between the TM1–2 and the β1-β2 loop [4]. This cascade leads to conformational changes in the TM1–2 loop, which moves toward the membrane domain. The motion is supported by contacts between Lys53_I_ and Glu375_H_. [6] These conformational changes also lead to the formation of ion pairs between TM1–2 (MbhI), PSST (MbhJ), and TM5–6 (MbhM) loops. The interaction of the TM1–2 loop with the MbhH subunit via Lys53_I_-Glu375_H_ is shown, together with key residues along the putative proton pathway in MbhH.

Despite some catalytic similarities to soluble hydrogenases,^14,15,23^ Mbh also shows notable differences in its proton transfer mechanisms (Figure 5A). Two distinct proton transfer pathways are identified in Mbh that are operational based on the [NiFe] redox state. Our results suggest that the first proton transfer (in the Ni–SI_a_ → Ni–L transition) takes place via Glu21_L_ (Glu pathway), while the second proton (Ni–C →Ni–R transition) can occur either via Glu or the His (His75_L_) pathways. Remarkably, the reaction barrier for the proton transfer between Glu21_L_ and Cys374_L_ during the Ni–C → Ni–R transition is higher (7.7 kcal mol^–1^) than that in the Ni–SI_a_ → Ni–L transition (2 kcal mol^–1^) (Figures 2D and 3A). However, the barrier decreases (by *ca*. 5 kcal mol^–1^) in the presence of a protonated His75_L_ due to electric field effects (Figure S25). These findings suggest a possible interplay between the two proton transfer pathways in facilitating the delivery of the second proton to the active site. Previous experiments on the soluble [NiFe] hydrogenase from *Pyrococcus furiosus* found large kinetic isotope effects (KIEs) of the Ni–SI_a_ →Ni–C conversion (via Ni–L) in the range of 6–43.^38^ Given that Cys374_L_ and Glu21_L_ share a strong hydrogen bond, the high KIEs suggest that quantum nuclear effects could play an important role in the proton transfer reaction, in addition to the low reaction barrier of 2 kcal mol^–1^ (Figure 2D) that also supports a fast transition.

It is to be noted that the coordination environment around Ni and Fe in different catalytic states could also play a critical role in the activation of the two proton pathways. Specifically, in the Ni–L state, we observed a strong coordination between Ni and S (Cys377_L_), with a bond length of 2.30 Å, while in the Ni–R state, this bond length increased to 2.74 Å, while the Ni–S (Cys374_L_) bond distance remained nearly identical in both states. This suggests a weaker binding of Cys377_L_ to the Ni in the Ni–R state as compared to the Ni–L state, indicating that it could tune the p*K*_a_ values of residues and possibly alter the ligand field environment to facilitate proton transfer along the His pathway during the Ni–C → Ni–R transition. The activity of the Glu pathway during Ni–C →Ni–R is likely to also depend on the conformation of the β1-β2 loop, which harbors Glu21_L_, and could function as a coupling element during the redox-driven proton pumping.

In soluble hydrogenases, both protons could be transported via the Glu pathway.^15^ In this regard, Evans *et al.*(56) studied the role of Glu28 (Glu34 and Glu21_L_ in *Dv*MF and Mbh, respectively) in the O_2_-tolerant [NiFe] Hyd1 from *Escherichia coli* during H_2_ oxidation by using an E28Q mutant, which accumulated mainly the Ni–R and Ni–C states. These findings led to the conclusion that Glu28 is not essential for the proton transfer during the transition from Ni–R to Ni–C, while the residue was found to be critical in the subsequent Ni–C → Ni–SI_a_ transition. These results are consistent with our work, in which we suggest that the first proton originates exclusively from the “Glu” pathway, while the second proton could be transported along either pathway. The two proton transfer pathways in Mbh thus show a unique resemblance to the proton transfer linked to Q reduction in Complex I, where both His38^Nqo4^ (part of the β1-β2 loop) and Tyr87^Nqo4^ function as the likely proton donors.^40^

We note that several structural models for the Ni–L^47,48^ and Ni–R^23,57,58^ states have been reported based on spectroscopic signatures, indicating different structural isomers. For instance, temperature-dependent FTIR studies of the soluble [NiFe] hydrogenases observed protonation/deprotonation of the residue homologous to Cys374_L_ (Cys546 in *Dv*MF) with an Δ*H* and Δ*S* of 1.5 ± 0.8 kcal mol^–1^ and 6.1 ± 10.3 kcal mol^–1^ K^–1^, respectively, suggesting that the cysteine residue can indeed undergo protonation change in the Ni–L state.^47^ This compares well with our finding on exergonic proton transfer from Glu21_L_ to Cys68_L_ or Cys374_L_ in the Ni–L state. Similarly, a recent IR and EPR investigation on a regulatory [NiFe]-hydrogenase from *Cupriavidus necator* proposed that the interconversion between the Ni–L_1_ and Ni–L_2_ forms does not require the breaking of covalent bonds,^48^ which is consistent with our finding of two isoenergetic rotamers (**3Y2** and **3B**) of the protonated Cys374_L_ in the Ni–L state (Figure 5A). Interestingly, Greene *et al.*(59) found based on the FTIR and absorption spectroscopic experiments on a soluble hydrogenase from *Pyrococcus furiosus*, two distinct forms of Ni–L. Our work thus further corroborates these findings on the active participation of different degenerate forms of Ni–L in Ni–SI_a_ → Ni–C transition, and provides a structural assignment for these states. Similar to the Ni–L state, at least three unique spectroscopic signals have been identified for the Ni–R state, implying the coexistence of multiple metastable species. Based on our findings, these states could arise from the different rotamers of the protonated Cys374_L_ (**5E2**^**LS**^ and **4B**^**LS**^), from the different H_2_ binding modes, and/or the dihydride-bound state (**4B1**^**LS**^ and **4D**^**LS**^) (Figure 5A). Our work thus provides a structural proposal for these spectroscopically characterized states and further highlights the involvement of several spin and conformational states during the catalytic cycle.

As discussed above, our study reveals striking similarities between Mbh and respiratory Complex I, such as conformational changes in the β1-β2 loop, the TM1–2 (MbhI), TM5–6 (MbhM), and the PSST loop (MbhJ) that are conserved within the superfamily, while the MbhH subunit is homologous to the antiporter-like Nqo12 subunit of Complex I. We suggest that the β1-β2 loop plays a critical role in H_2_ catalysis by shuttling protons from the bulk toward the [NiFe] active site, while enabling a redox signal propagation toward the membrane domain that triggers ion pumping across the membrane.^5^

The long-range signal transduction between the electron transfer activity in the hydrophilic domain and the ion transport in the membrane domain could be achieved through the redox state of the [NiFe] cluster and the protonation state of Cys374_L_ and Glu21_L_. For each catalytic turnover, the conformational changes could be triggered during the Ni–C → Ni–R transition via motion in the network of loops (Figure 5B). The primary coupling event could involve an outward flip of Glu21_L_ and the subsequent contraction of the β1-β2 loop, which further leads to changes in the ion pairs in TM5–6 (MbhM), PSST (MbhJ), and the TM1–2 loop (Figure 4H). We note the TM1–2 loop shows a lower sequence conservation (ca. 20%) relative to the same region in Complex I (Figure S31). In this regard, we note that Asp379_L_ is conserved in Mbh and Complex I, but not present in hydrogenases, suggesting that the Lys24/Asp379 interaction of MbhL could be important for energy transduction (Figure 4E). In contrast, Glu49_I_ is unique for Mbh (Figure 4F) and forms an ion pair with Lys40_L_, while MbhM (NuoH in Complex I) comprises the unique Glu216_M_ that interacts with Arg64_J_ (Figure 4G). The identified electrostatic network provides an important basis for future mutagenesis studies.

## Conclusions

The membrane-bound hydrogenase (Mbh) from *Pyrococcus furiosus*, a member of the Complex I superfamily, serves as an intriguing system for studying the interplay between [NiFe]-enabled H_2_ catalysis and ion transport and its evolutionary relation to Complex I, which catalyzes quinone reduction. By combining density functional theory (DFT) with correlated *ab initio* methods and atomistic molecular dynamics simulations, we derived key insight into the molecular mechanism of H_2_ evolution in Mbh, which has so far been missing, together with a detailed analysis of energetics and dynamics linked to this process. Our findings on the [NiFe] catalysis provide insight into the structures of the Ni–L and Ni–R states,^23,48^ the involvement of various spin states during catalysis, as well as a rationale for the activation of the Glu and His proton pathways^56^ that is likely to apply also for soluble [NiFe] hydrogenases.

We suggested that Mbh employs redox-driven conformational changes similar to those of Complex I, particularly around the conserved β1-β2 loop that could be mechanistically important for transducing the redox energy into ion transport across the membrane. Moreover, we suggest that Mbh employs electric field effects formed by redox and protonation changes–a mechanism that could serve as a general energy transduction principle in nature.

## Materials and Methods

### Quantum Chemical Models

Quantum chemical DFT models were built to probe the geometric and electronic structure of the [NiFe] active site of Mbh. The DFT models were built by combining conserved parts of a high-resolution X-ray structure (at 0.89 Å resolution, PDB ID: 4U9H) of the soluble [NiFe] hydrogenase from *Dv*MF, resolved in the Ni–R state,^36^ together with our MD-relaxed system of Mbh,^5^ where we substituted all residues that are unique for Mbh (Figure 1B). The central core of the model contained Fe, Ni, the hydride, two CN- and one CO ligands, and all residues in the first and second coordination spheres (Asn36_J_, Glu21_L_, Cys68_L_-Gly69_L_-Ile70_L_-Cys71_L_, His75_L_, Ser110_L_, Ala318_L_-Pro319_L_-Arg320_L_, Leu323_L_, His325_L_, Glu343_L_-Pro344_L_-Thr345_L_, Asp372_L_, Cys374_L_-Leu375_L_-Ser376_L_-Cys377_L_, and a water molecule) (Figure 1B). The residues with a truncated backbone were terminated by a methyl group (−CH_3_) at the C_a_ atom. The C_a_ atoms and the linker hydrogen atoms of residues were fixed at their X-ray positions during the geometry optimization. The model contains a total of 264 atoms. All geometry optimizations (ground state, transition state, product states) were performed at the TPSSh^60^ level with the central core of the active site comprising of Fe, Ni, S, two CN- and CO assigned def2-TZVP basis set,^61^ while the rest of the atoms were described with the def2-SVP basis sets. We also applied the multipole accelerated resolution of identity (MARI-J) approximation^62^ during the optimization protocol, whereas dispersion effects were included using the empirical dispersion correction with Becke-Johnson damping (D3-BJ).^63^ A higher DFT integration grid (m4) and tighter SCF thresholds were used throughout (scfconv 8) in all computations. We also employed the implicit solvation COSMO scheme^64,65^ with the dielectric constant set to 4.0. The molecular Hessian used for estimation of free energies was computed numerically at the TPSSh/def2-TZVP/def2-SVP level of theory (same as optimization protocol), with scaling factors (0.9615) of the vibrational frequencies based on
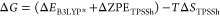


The reaction energetics reported in this work (see Figures 2,3) are based on the equation shown above, unless stated otherwise. The reaction pathways were optimized between reactant and product states using a method related to the zero-temperature string method, as implemented in TURBOMOLE 7.5.1.^66^ The final single-point energy calculations were performed with B3LYP* (with 15% Hartree–Fock exchange) and scalar relativistic effects were included using the exact two-component (X2C) Hamiltonian.^67−70^ We used x2c-TZVPP for Fe and Ni, and x2c-TZVP basis set^71^ for the rest of the atoms. The choice of B3LYP* functional was chosen based on its performance against the random phase approximation^72^ (RPA) method and domain-based pair natural orbital coupled cluster with singles, doubles, and full iterative triples (DLPNO–CCSD(T_1_))^73−75^ calculations to predict spin-state energetics accurately. The final single-point energy and molecular Hessian calculations were performed on a DFT model (Figure S4). The results of the benchmark (geometry, spin state, and reaction energetics) are shown in the Supporting Information. All the calculations were performed with the TURBOMOLE v. 7.5.1.^66^

The electric field along the proton transfer pathway was computed using optimized structures at the TPSSh/def2-TZVP/def2-SVP level of theory with the TURBOMOLE v. 7.5.1 suite. The electric field contributions of the water molecule between Glu21_L_/Cys374_L_ were omitted from the DFT models.

### Molecular Dynamics Simulations

Atomistic molecular dynamics simulations of the Mbh (PDB ID: 6CFW)^1^ model were performed using the CHARMM36 force field for protein/lipids and water. Parameters for the FeS clusters and [NiFe] in different catalytic states (see Tables S22, S23) were derived from in-house DFT calculations (https://github.com/KailaLab/ff_parameters), with the remaining system treated using the CHARMM36m force field.^76^ The system was embedded in a 1-palmitoyl-2-palmitoleoyl-*sn*-glycero-3-phosphoinositol (PYPI) membrane using CHARMM-GUI^77^ modeling unresolved side chains,^5^ as well as different protonation states with lipids in the cleft between MbhM and MbhH. The model was embedded in a 200 × 100 × 168 Å^3^ box comprising TIP3P water molecules and ions to mimic a 250 mM NaCl concentration. The [NiFe] catalytic site was modeled into Mbh using the high-resolution soluble hydrogenase (PDB ID: 4U9H).^36^ Initial protonation states were obtained from Poisson–Boltzmann electrostatic calculations with Monte Carlo sampling,^5^ based on models with the cofactors treated in their oxidized state (see SI Appendix, Table S24, for a list of residues with nonstandard protonation state). All MD simulations were performed in an *NPT* ensemble at *T* = 310 K and *p* = 1 atm, using a 2 fs integration time step, and with electrostatics modeled using the particle mesh Ewald (PME) method. The system was gradually relaxed for 10 ns with harmonic restraints of 2 kcal mol^–1^ Å^–2^ on all protein and cofactor heavy atoms, followed by 10 ns with harmonic restraints of 2 kcal mol^–1^ Å^–2^ on all protein backbone heavy atoms, and by 10 ns equilibration with weak (0.5 kcal mol^–1^ Å^–2^) restraints on all C_α_ atoms and 0.5 μs production runs. All classical MD simulations (3.5 μs in total) were performed using NAMD2 (v. 2.12/2.13) for equilibration and NAMD3^78^ for production runs. The simulations were analyzed using VMD.^79^ Multiple sequence alignment (MSA) for soluble hydrogenases and the Complex I superfamily was done with ClustalW^80^ and visualized with Jalview.^81^

## Data Availability

Cartesian coordinates of all quantum cluster DFT models (115 structures) are deposited in the Zenodo repository (https://zenodo.org/records/10823885). The force field parameters for the [NiFe] center are available at https://github.com/KailaLab/ff_parameters.

## Supporting Information Available

Extended methods, geometric and molecular energetics benchmark, MD simulation data, DFT models, reaction profiles, geometric analysis, additional information of [NiFe] catalysis (PDF)
Movie S1 (MPG)
Movie S2 (MPG)
Movie S3 (MPG)

## Supplementary Material

ja4c04476_si_001.pdf

ja4c04476_si_002.mpg

ja4c04476_si_003.mpg

ja4c04476_si_004.mpg
